# Users’ Responsiveness to Persuasive Techniques in Recommender Systems

**DOI:** 10.3389/frai.2021.679459

**Published:** 2021-07-08

**Authors:** Alaa Alslaity, Thomas Tran

**Affiliations:** University of Ottawa, Ottawa, ON, Canada

**Keywords:** recommender systems, recommender-user interaction, persuasive principles, personalized persuasive, Cialdini's principles, user modeling

## Abstract

Understanding user’s behavior and their interactions with artificial-intelligent-based systems is as important as analyzing the performance of the algorithms used in these systems. For instance, in the Recommender Systems domain, the *accuracy* of the recommendation algorithm was the ultimate goal for most systems designers. However, researchers and practitioners have realized that providing accurate recommendations is insufficient to enhance users’ acceptance. A recommender system needs to focus on other factors that enhance its interactions with the users. Recent researches suggest augmenting these systems with persuasive capabilities. Persuasive features lead to increasing users’ acceptance of the recommendations, which, in turn, enhances users’ experience with these systems. Nonetheless, the literature still lacks a comprehensive view of the actual effect of persuasive principles on recommender users. To fill this gap, this study diagnoses how users of different characteristics get influenced by various persuasive principles that a recommender system uses. The study considers four users’ aspects: age, gender, culture (continent), and personality traits. The paper also investigates the impact of the context (or application domain) on the influence of the persuasive principles. Two application domains (namely eCommerce and Movie recommendations) are considered. A within-subject user study was conducted. The analysis of (279) responses revealed that persuasive principles have the potential to enhance users’ experience with recommender systems. The study also shows that, among the considered factors, culture, personality traits, and the domain of recommendations have a higher impact on the influence of persuasive principles than other factors. Based on the analysis of the results, the study provides insights and guidelines for recommender systems designers. These guidelines can be used as a reference for designing recommender systems with users’ experience in mind. We suggest that considering the results presented in this paper could help to improve recommender-users interaction.

## Introduction

Artificial intelligence and machine learning techniques have become essential for a wide variety of applications. One of the commonly applied systems nowadays is *Recommender System* (RS), which is the software that suggests items (such as information, products, services, etc.) based on user’s preferences (Jannach et al., 2010). The recommendation process relies on various techniques, such as machine learning and Natural Language Processing. Recommender systems have been deployed in many domains, such as health, learning, movie, music, and eCommerce. Since the emergence of RSs, there was a significant focus on improving the prediction capability and the *accuracy* of these systems. Later on, researchers realized that an accurate recommendation does not necessarily imply a better user experience (McNee et al., 2006; Knijnenburg et al., 2012). Recommender systems should also emphasize other factors that enhance the interaction between recommender systems and their users. One of the recently emerged research directions that consider this need fosters the idea of adopting human-related theories from the social sciences domain, such as persuasiveness of social communication, into the recommendation domain.

Although computers do not have the same communication skills as humans, researchers suggest that human-human interactions’ theories can be adopted for human-computer interactions (Yu et al., 2011). According to Fogg (2002), users can be more persuaded by systems that have human-like features. Therefore, the author emphasized the importance of understanding the social role of computer systems that take the role of advising users (such as the case of recommender systems). According to Yoo et al. (2012), traditional persuasive principles could provide a useful framework to study the communication between RS (as systems that advise users) and its users. That is, integrating persuasive features to the recommendations could enhance the communication between RSs and their users. In movie recommenders, for instance, accompanying the recommended movie with a persuasion cue (e.g., your friends X, Y, and Z, also watched the recommended movie) has the potential to enhance users’ trust in the RS.

Researchers have recently started investigating how users respond to (or interact with) different persuasive principles. Despite the increasing research interest in this direction, the work is still limited, especially in the area of recommender systems. A limited number of researchers studied how users interacted with different persuasive principles, considering the latter’s different characteristics. These limited studies focus on the impact of users’ characteristics while neglecting other important factors, such as the application domain (or the context). Also, most of the studies (such as Alkis and Temizel, 2015; Oyibo et al., 2017a) are not designed in particular for the RS area. Besides, most of these studies involve a limited sample in the sense that they consider participants from a single culture (e.g., Alkis and Temizel, 2015; Gkika et al., 2016; Oyibo et al., 2017b), a particular community, such as university students (e.g., Gkika et al., 2016), or limited age range (e.g., Alkış and Temizel, 2015).

This paper aims to fill these gaps and mitigate the limitations by investigating how recommender-user interaction is affected by the six persuasive principles of Cialdini (Cialdini, 2001), which are reciprocity, Commitment, Social Proof, Liking, Authority, and Scarcity. In particular, the paper presented a user study that examines RSs users’ responsiveness to Cialdini’s persuasive principles and the extent to which users’ attributes and the application domain affect this responsiveness. To achieve our goal, we conducted an online questionnaire that consists of two main parts: the personality test part and the persuasion test part. The persuasion test part, in turn, is divided into three subparts: the eCommerce domain, the movie domain, and the general (no domain) parts. In each part, participants were asked to rate six sentences that represent Cialdini’s persuasive principles. More details about the design of this user study are discussed in *The Study*. Based on the data analyses, the paper provides general guidelines for designing persuasive RSs. Our results indicated that Cialdini’s principles influence the decisions of RS users. The influential degree of these principles is affected by users’ characteristics, especially the culture and personality traits. Besides, the impact of users’ characteristics on their responsiveness to the persuasive principles varies from one recommendation domain to the other.

This study is different than the current literature in various aspects:

1) it is designed and tailored to the recommendation systems domain.
2) It investigates the effect of multiple factors, including the user’s age, gender, culture, and personality traits, in addition to the persuasion context presented by the application domain.
3) It explores the impact of each of the users’ characteristics in conjunction with the application domain factor.
4) The study sample is relatively heterogeneous in the sense that it involved participants from different continents, ages, and gender.

The analysis and the guidelines presented in this paper contribute to the literature in different ways. First, it provides a user profiling; the study defines how different persuasive principles influence a group of users (based on different characteristics). Second, these results serve as a reference for persuasive RSs designers to tailor their design to achieve users’ preferences. Based on the results, a designer can provide multiple implementations (each targeting different users’ groups) of the same system. Third, many RSs use explanations to clarify why a particular item is recommended to the user (Gkika and Lekakos, 2014a). These explanations can be more effective if they encompass persuasion cues. By utilizing the guidelines presented in this paper, designers can personalize explanations. Personalizing explanations, in turn, makes them more helpful and influential. Forth, designers can also utilize our results to enhance recommender-user interactions by providing more influential interfaces. For instance, a persuasive RS may present the list of recommended items in various ways. For a user who is highly influenced by the *Authority* persuasive principle, the list can be presented with a picture for an authority beside it. On the other hand, for a user who is highly influenced by the *Social Proof* principle, the list can be presented along with the number of users who used that recommendation.

The rest of this paper is organized as follows: *Background and Related Work* discusses the background and reviews the literature. *The Study* discusses the study methodology. The results are displayed and analyzed in *Data Analyses and Results*. A discussion and some guidelines are provided in *Discussion and Design Guidelines*. Finally, the paper is concluded in *Conclusion and Future Work*.

## Background and Related Work

This section explains the mains concepts related to our work. Specifically, it introduces personality traits and Cialdini’s persuasive principles. Then it explores the related work.

### Personality Traits

User personality can be defined as “a set of characteristics possessed by a person that influences his or her cognitions, emotions, motivations, and behaviors in various situations” (O’keefe, 2002). Psychologists have set up various theories about human personalities. One of the most common personality theories is the Five-Factor Model (FFM). It was initially introduced in 1961 by Tupes and Christal (1992). The FFM is a hierarchical organization of human personality traits that contains five wide dimensions, which are also known as the Big Five (BF) personality traits.^1^ Each dimension represents a set of frequent interpersonal situations that construct patterns. These patterns are known as relatively consistent, and they characterize an individual’s life (Sullivan, 2013). Therefore, they distinguish one personality from the other, and they represent the differences between personalities (Soto and Oliver, 2009).

The big five personality traits are *Openness*, *Conscientiousness*, *Extraversion*, *Agreeableness*, and *Neuroticism*. Each dimension comprises many facets that distinguish it from the other four dimensions. Table 1 lists the five personality traits, along with their characteristics defined based on the study of McCrae and Oliver (1992). It is worthwhile to mention that the FFM does not indicate that the differences between personalities can be confined to five traits. However, they represent a broader abstraction of the personality with several more specific characteristics (John and Srivastava, 1999). The FFM does not classify people on a zero/one basis. Instead, every one of the five traits can be presented as a spectrum, where individuals may fall anywhere on each spectrum.

**TABLE 1:** The big five personalities.

Factor	Adjectives
Openness	Artistic, curious, imaginative, insightful, original, and wide interest
Conscientiousness	Efficient, organized, planful, reliable, responsible, and thorough
Extraversion	Active, assertive, energetic, enthusiastic, outgoing, and talkative
Agreeableness	Appreciative, forgiving, generous, kind, sympathetic, and trusting
Neuroticism	Anxious, self-pitying, tense, touchy, unstable, and worrying

This study considers the FFM because it is the most widely accepted personality model (Lambiotte and Michal, 2014), and it is a well-established model (Dunn et al., 2009). Also, this model categorizes all people into five dimensions only. These five factors can describe the wide range of personalities (Ewen and Robert, 2014), and its comprehensiveness has been confirmed theoretically and practically (Ryan and Edward, 2000; McCrae and Oliver, 1992). Finally, the FFM can be quantitatively measured; therefore, it is suitable for usage in recommender systems.

Several tests have been proposed to assess an individual’s personality. One of the most popular questionnaires is the Big Five Inventory (BFI-44) (Rammstedt and Oliver, 2007; John et al., 2008). It is known as BFI-44 because it consists of 44 short-phrase items. These 44 items are rated on a five-step scale from 1 (Strongly disagree) to 5 (Strongly agree). Then, based on particular equations, the individual’s rank on each personality trait is calculated. Our study relies on the BFI-44 because it is a well-established measurement of personality traits (Tkalcic and Lwe, 2015). It is also a short version of the most accurate inventory in the literature called NEO-Personality-Inventory-Revised (NEO-PI-R) (Costa and McCrae, 2008). The BFI-44 is preferred over the NEO-PI-R, especially when the test time is limited, as in the RS domain.

It is worth mentioning that many super short inventories have been introduced to mitigate the limited assessment time issue. For instance, the BFI-10 is an abbreviation of the BFI-44 instrument that consists of ten items only (Rammstedt and Oliver, 2007). BFI-10 requires less time than the BFI-44, but we did not consider it because there are always tradeoffs between shorter and more accurate assessments; the more the number of questions is, the more accurate the assessment is Nunes et al. (2012). Given that the standard BFI contains only 44 short phrases and takes 5 min on average, it is not recommended to use the shorter version unless there are exceptional situations (John et al., 2008).

### Persuasive Principles

Behavioral scientists and practitioners have proposed different taxonomies of persuasion strategies. Oinas-Kukkonen and Harjumaa (2008) developed 28 persuasive systems design tools, which are built on the strategies developed by Fogg (2002). The developed techniques were categorized into four categories, which are Primary task (which includes seven principles that support the carrying out of the user’s primary task), Dialogue (includes design principles related to the implementation of computer-human dialogue support), System Credibility (encompasses principles that concern how to design a system so that it is more credible and persuasive), and Social Support (which indicates principles that support the design of systems that motivates users by leveraging social influence).

Halko and Kientz (2010) surveyed the literature and declared eight persuasive strategies as the main strategies used in persuasive computing. They categorize these strategies into four general approaches. Each approach compromises two complementary tactics. The four categories are Instruction Style (Authoritative, Non-Authoritative), Social Feedback (Cooperative, Competitive), Motivation Type (Extrinsic, Intrinsic), and Reinforcement Type (Negative, Positive). Another taxonomy was proposed by Cialdini (2001). This taxonomy defines six persuasive principles known as the six weapons of influence. These principles are Reciprocity, Scarcity, Authority, Social Proof, Liking, and Commitment.

Other taxonomies have also been discussed in the social sciences literature. Due to the extensive research done in the theoretical part of this direction, the large number of strategies cannot be exhausted in one study. Thus, we will not dive deeper into discussing these taxonomies as it is out of the scope of this work.

Among the wide range of persuasive principles, this article deploys Cialdini’s six principles because they have been widely used in the literature and social scientists verified them as global persuasive approaches (Gkika et al., 2016). Besides, these principles are simple and easy to implement, so they have been used by other studies in the RS domain (Gkika et al., 2016).

The six persuasive principles, along with their definitions (Cialdini, 2001), are:

• *Reciprocity*: “People repay in kind.” Individuals tend to return a favor. Accordingly, individuals are more motivated to accept recommendations from a person they are in debt to. For instance, if a friend sent you a birthday gift, you feel obligated to give her a gift on her birthday. Another example is when you’re planning to visit a friend who visited you previously. Many computerized systems deploy this principle such that they offer new users free subscriptions for a test period or giving a discount upon registration.
• *Scarcity*: “People want more of what they can have less of.” People consider scarce items as more valuable. A common example of this principle is when stores mentioned a limitation of an item. This principle is widely used especially in sales settings. Most well-known eCommerce websites, such as Amazon, deploys this principle by offering limited-time promotions or displaying the number of items left in the stock. It is also used by other platforms, such as room booking (e.g. Airbnb), when they show statements like “*rooms in XYZ are in a high demand*.”
• *Authority*: “People defer to experts.” People’s acceptance of a suggestion or a recommendation increases when a legitimate authority makes it. For instance, it is more likely to buy toothpaste if a well-known dentist recommends it. Using titles, such as Prof, Doctor, CEO, etc., with headlines and blog posts is another example of deploying Authority principles. In software systems, this principle is deployed in various ways. A common way is to present experts' opinions or reviews about items.
• *Social Proof:* “People follow the lead of similar others.” When people are uncertain, they rely on the actions of others to decide. So, people have a tendency to do what others do. For instance, if an individual plans to reserve a hotel room, she usually checks out the hotel's reviews. The number of followers on social media, customers reviews, and mentioning the company in blog posts are examples of implementing Social proof in online businesses, such as eCommerce recommender systems.
• *Liking*: “People like others who like them.” People are most likely accepting requests made by somebody they like. The Liking principle is connected, to some extent, with the Authority and Social Proof principles but it concerns arguments from people we know in real life. For instance, an individual may favor one store over the other only because she likes its employees. In computerized systems, it is also essential to ensure that the users enjoy the service. So, the service or product should be presented in an attractive way.
• *Consistency* (or *Commitment*): “People align with their clear commitment.” People feel obligated to their previous opinion or behavior. The basis of this principle is that if people commit to doing small requests, it will be easier to convince them to do larger requests. Commitment leads to customers’ loyalty. In online marketing, for example, the system simplifies the signing up process to let users sign up (which indicates the initial commitment). Another example is when giving the user a period during which she can ask for a full refund.

These principles explain people’s tendencies to comply with a request; they provide means that cause one person to say yes to another one. Implementing these principles appropriately can increase the acceptance of the advice, requests, or recommendations (Alslaity and Tran, 2020b). Therefore, using these principles in this article aims to examine how users’ attitudes can be changed.

The essence behind these principles and other persuasive principles is that individuals vary in their responses to the persuasive attempts. That is, the one-size-fits-all approach is not accepted for influsencing people. So, these principles describe different ways of persuasion. It is worth mentioning that selecting the most suitable strategies for a specific domain is often based on the designers’ intuition (Orji et al., 2014). Thus, we decided to choose Cialdini’s persuasive principles because they are simple yet general enough so they can be deployed in a wide array of applications. This does not imply that other persuasive principles are not important or cannot be used in the RS domain.

### Related Work

This section discusses previous researches that are most related to our work. It explores the existed work in personalizing persuasive principles with a focus on the recommender systems domain. In particular, we articulate the search queries according to the following questions:

• Question 1: What work was done that discusses the impact of RS users’ characteristics on their responsiveness to Cialdini’s persuasive principles?
• Question 2: What work was done that discusses the effect of the RS context on the influence of Cialdini’s persuasive principles?

The work that discusses the effect of persuasive principles (such as Cialdini’s six principles) in the context of recommender systems is relatively limited (Gkika and Lekakos, 2014a). However, the research in this direction is gaining increasing attention. As a result, some approaches have been introduced in various areas of RSs, as follows:

Gkika and Lekakos (2014a, 2014b) investigated the feasibility of using Cialdini’s persuasive principles as explanations in RSs. Particularly, they investigate the effect of using these principles on users’ intention to use a recommendation. The study comprises two parts. In the first part, the authors designed persuasion explanations. They suggested 30 different explanations, five for each influence strategy. Then, seventeen experts were asked to select the best matching explanation for each strategy. In the second part, the authors conducted a user study. They deployed a prototype of movie RS, and they included the designed explanations along with each recommendation. The participants were asked to rate each explanation in terms of its impact on their decision to watch the movie. One hundred eighty-four subjects participated in the study. The main conclusion of this study is that incorporating persuasive explanations to recommendations that are close to users’ preferences will affect their behavior in terms of accepting/rejecting recommended items.

#### Demographics and Persuasive Principles

This section concerns studies that study the impact of users’ demographics on the effectiveness of different persuasive principles. Age, gender, and culture are among the most commonly discussed demographic information. Following are studies that

Regarding the relationship between users’ characteristics and Cialdini’s principles, researchers consider various users’ characteristics such as age, gender, and personality traits. Oyibo et al. (2018a), for instance, conducted a study that aims to determine Africans’ (Nigerians, in particular) persuasion profiles based on Cialdini’s principles. The authors also investigated whether gender influences the responses of the participants. Eighty-eight participants responded to the study. The results indicated that the six persuasive principles are influential for the Nigerians but at different levels. In particular, the results revealed that *Commitment*, *Reciprocity*, and *Authority* are the most influential principles, followed by *Liking*, *Consensus*, and finally *Scarcity* as the least influential. The study also concluded that males were found to be more susceptible to Commitment and Authority than females among Nigerians.

In another study, Oyibo et al. (2018b) investigated the impact of personality on users’ responsiveness to Cialdini’s principles and how it is affected by cultural backgrounds. In particular, the study considered two cultures, which are Nigerians and Canadians. A user study with 248 responses (88 Nigerians and 196 Canadians) was conducted. The results revealed that Nigerians’ responsiveness is different from the Canadians for all strategies except for the *Commitment* strategy. Specifically, *Authority* and *Scarcity* were found to be the most effective on Nigerians. On the other hand, *Reciprocity* and *Liking* were found the most effective on Canadians.

Alslaity and Tran (2020a) investigated the effect of RS’s domain on users’ responsiveness to Cialdini’s persuasive principles. The authors conducted a user study that consists of two sections. One represents the domain of eCommerce recommendations, while the other represents movie recommendations. In each section, the participants were revealed to six persuasion statements; each one represents a principle of Cialdini’s principles. Participants were asked to rate each statement based on its impact on their decision to buy an item or watch a movie. One hundred seven participants were responded to the study. The results demonstrated that the application domain has an impact on the effect of persuasive principles. So, it should be considered when designing a persuasive RS.

#### Personality Traits and Persuasive Principles

Regarding the relationship between personality traits (namely, the FFM) and Cialdini’s persuasive principles, the literature has recently witnessed increased research in this direction. Nonetheless, the research in this direction is still relatively limited (Oyibo et al., 2017a), especially in the area of RSs. To the best of our knowledge, only four prior studies have discussed this correlation, as follows.

Oyibo et al. (2017a) also studied the relationship between the Big-Five personality traits and Cialdini’s persuasive principles. The study was conducted in Canada. It used a 32-item scale to measures participants’ responsiveness to Cialdini’s principles. A total of 216 responses were considered in the study; all of them are Canadians. The results revealed that *Neurotic* participants are the least predictor of the persuasive principles, as they only predict *Social Proof*. *Conscientious*, *Agreeable,* and *Open* participants turn out to be the most consistent predictors of Cialdini’s principles. Also, they noticed that none of the personality traits predicts *Scarcity* among Canadians.

Alkış and Temizel (2015) conduct a similar study. This study is similar to the study of Oyibo et al. (2017b) such that it discusses the relationship between personality traits and Cialdini’s principles. However, this study considered a different sample, which includes 381 participants; all of them are Turkish undergraduate students. To collect data, the study conducted a structured questionnaire that comprised the Big Five personality traits scale and the Responsiveness to Persuasion Strategies scale. The study found that personality traits are important in selecting influence strategies, and *Agreeable* people were found to be the most susceptible to Cialdini’s principles compared to the other traits.

In the RS domain, Gkika et al. (2016) discussed the interaction between Cialdini’s persuasive principles and RS’s users’ personalities. The BFI-44 was used as a measure of users’ personalities. The authors conducted a within-subject user study that exposed the participants to six different explanations (one for each persuasive principle). The participants were asked to rate the recommendations in order to evaluate which principle, if there is any, influenced the user's intention to watch a movie. The results found that *Authority* and *Social Proof* are the most effective principles. Besides, the results indicated that users’ personality impacts the effectiveness of persuasive methods.

In a recent study, Alslaity and Tran (2020b) also examined how RS’s users’ interaction with the persuasive principles is affected by their personalities. The study relied on the Five-Factor Model to distinguish users’ personalities. A user study with a more heterogeneous sample (comparing to Gkika et al.’s study) was conducted to test users’ persuadability. The data collected from 279 participants shows that personality traits could affect RS’s users’ responses to persuasive principles. However, the authors suggest that users’ personalities should be considered with other factors, such as the application domain.

To summarize, the literature review revealed increasing attention toward personalizing persuasive principles. The existed work revealed some conclusions in this direction. Nonetheless, there is still a huge untapped potential to maximize the impact of persuasive applications (Berkovsky et al., 2012). Based on the literature review, we can summarize the major gaps and limitations that we try to fill or mitigate by the following points:

• Most of the existed studies focus on the impact of a *single factor* (e.g., culture or gender) while ignoring other important factors. Also, the impact of the studied factors is discussed *in isolation* of other factors that may have a significant effect if the interaction effect is considered.
• The work in the relationship between the Big Five personality traits and Cialdini’s principles is *still limited*, especially in the area of RSs. Only three studies (Oyibo et al., 2017b; Alkış and Temizel, 2015; Gkika et al., 2016) investigated this relationship. *Only two* of these studies (Gkika et al., 2016; Alslaity and Tran, 2020b) have addressed this topic in RS's context.
• Many of the studies are *generic*, such that they are not designed to a particular application domain or context. According to Oyibo et al. (2018a), the actual users’ responsiveness to the persuasive principles may differ when implemented and evaluated for real persuasive application. In our study, we tailored the persuasion questionnaire to the recommendation domain.
• They have a *limited study sample* such that the number of participants is relatively small, or the participants are homogeneous (e.g., same age group, same culture, etc.).

Our user study aims to fill these gaps by investigating RSs users' esponsiveness to Cialdini’s principles, and the extent to which users’ attributes and the application domain affect this responsiveness. To achieve this, we conducted an online questionnaire that consists of two main parts: the personality assessment part and the persuasion test part. The persuasion test part, in turn, is divided into three subparts: the eCommerce domain, the movie domain, and the general (no domain) parts. In each part, participants were asked to rate six sentences that represent Cialdini’s persuasive principles. More details about the design of this user study are discussed next.

## The Study

Our user study's design and evaluation were guided by the Goal, Question, Metric (GQM) framework of Basilwe (1992). The GQM is a systematic approach that is commonly used in software engineering, and it can be adopted in different settings (Azham and Ferneley, 2008), such as behavioural change and gamification (Tuah and Wills, 2020). Following this framework, we first identified the goals of the study. Then for each identified goal, we articulated a set of questions reflecting the goals and a set of measures to assess the achievement of the goals (more details are provided in *Data Analyses and Results*). The main goals of our user study are to:

• G1: Evaluate the persuadability of RS users and the impact of different factors on their responsiveness to persuasive principles. Specifically, The study considers the following factors: age, gender, culture, personality traits, and application domain.
• G2: Provide guidelines for user-centric (i.e., tailored to different users based on their characteristics) designing of persuasive recommender systems to enhance users-recommender interaction.

The following sections explain the study design and other components, such as the ethics approval, participants, data collection and analysis, and the results.

### Study Design

This study follows a within-subject study design; wherein, the same person tests all the conditions (i.e., all the questionnaire sections). The study starts by asking questions about the demographic background. Then, participants are required to answer two questionnaires, a personality questionnaire and a persuadability^2^ questionnaire. The first one is about assessing the personality traits according to the FFM, in which we deployed the BFI-44 items (discussed in *Personality Traits*). The second questionnaire (Persuadability^3^ questionnaire) aims to test users’ responsiveness to the six persuasive principles. This questionnaire is divided into three parts based on the application domain, as follows: 1) the general part, which does not reflect a particular domain. 2) eCommerce, which is designed to reflect the eCommerce RS domain. And 3) Movie part, whcih reflects the movie RS domain. In each part, we presented six different cues (or sentences); each cue represents one of Cialdini’s persuasive principles (as depicted in Table 2. The three parts of the persuadability questionnaire were ordered randomly to avoid biases in the results. Participants were asked to rate these sentences based on a seven-step Likert scale from −1 to 5, with −1 being the least level of responsiveness (or negative impact) and 5 being the maximum level.

**TABLE 2:** Persuasive sentences presented in the questionnaires.

Principle	Persuasive cue
Authority	*eCommerce RS*: The recommended item won three prizes as the best-manufactured product!
	*Movie RS*: The recommended movie won three oscars!
	*General*: Presenting an image of an expert uses the recommended item (ex: a doctor uses particular medication for his patients, or a security guard uses the recommended security lock)
Commitment	*eCommerce RS*: This item belongs to the kind of items you usually buy
	*Movie RS*: This movie belongs to the kind of movies you enjoy watching
	*General:* Using the “add to wish list” option. (“Wish list” contains items that you wish to buy in future)
Social proof	*eCommerce RS*: 87% of users rated the recommended item with 4 or 5 stars!
	*Movie RS*: 87% of users rated the recommended movie with 4 or 5 stars!
	*General*: Presenting the best sellers items
Liking	*eCommerce RS*: Your Facebook friends bought this item!
	*Movie RS*: Your Facebook friends like this movie!
	*General*: Well-designed (fancy and professional) website’s interface and product presentation
Reciprocity	*eCommerce RS*: A friend of you who bought the item you suggested to him/her in the past recommends you this item!
	*Movie RS*: A Facebook friend, who saw the movie you suggested to him/her in the past, recommends this movie!
	*General*: Giving you something for free (e.g., samples, gift, or free delivery)
Scarcity	*eCommerce RS*: The recommended item will be available for two months only!
	*Movie RS*: The recommended movie will be available for 2 months only!
	*General*: Display a countdown, beside an item, indicating the time remaining for an offer on that item

It is worthwhile to mention that the persuasive sentences that we used in the eCommerce and the movie parts of the persuadability questionnaire were inspired by and designed based on the study of Gkika et al. (2016). We adopted the same sentences for the movie RS as suggested by the authors; then, we followed the same convention to formulate persuasive statements for the eCommerce RS. The rationale behind adopting Gkika et al.’s methodology is that the persuasive statements used in their study were well-designed and thoroughly tested by seventeen experts to choose the best matching sentence for each persuasive principle. The study is also one of the few studies conducted for the RS context, which is the same as our context.

The questionnaires have a Likert scale, multiple choices, and open-ended questions. The Likert scale questions have a seven-step scale of graduation from −1 to 5, where each degree in the scale represents the extent to which each statement influences users' decisions (i.e., to accept or reject the recommendation). The seven scales' indications are as follows: 1 to 5 options are scaled from very low to very high positive effect, zero (0) is the neutral value, indicates no effect, and (−1) indicates a negative effect. The (−1) step is included because persuasion cues may cause resistance towards the recommendation; Kaptein and Dean (2012) found that persuasive strategies may not achieve their goals to influence users. Indeed, a negative response may be generated if the user receives an inappropriate message. For instance, a Scarcity strategy may have a negative effect on users who do not like to decide under stress.

The questionnaire was available online using *SurveyMonkey*,^4^ a well-known platform that provides online services to create and share surveys, collect responses, and analyze the results. *SurveyMonkey* is a secure, powerful, and intuitive website. Also, it offers a wide range of functionalities and services. Besides, the University of Ottawa has a university-wide license for the enterprise version of *SurveyMonkey*. By this license, most of the services can be accessed and used by the University of Ottawa staff for free.

Participants were asked to complete the questionnaire only once. To complete the study, we provided the participants with a link to the questionnaire. In the beginning, the participant should agree on the consent form to proceed and complete the other steps, which are: 1) demographic information, which includes age, gender, and country. 2) Personality test to specify their personalities according to the FFM. 3) Persuadability test, which consists of three sections. In each section, the participant needs to rate six persuasive statements and provide additional comments. Since the study does not use a real recommender system (i.e., no real recommendations were made), we introduced each section with a paragraph that puts the user in the context. The session takes approximately 15–20 min. The time factor was, sometimes, an obstacle towards the completion of the survey. To mitigate this issue, the questionnaire was available 24 h a day, and the participants were given a choice to complete the questionnaire at their own convenience.

### Ethics Approval

For this study to be compliant with the Canadian ethical standard, an application is submitted to the Research Ethics Board (REB) at the University of Ottawa. The REB helps ensure that this research meets the highest ethical standards and that the greatest protection is provided to participants who serve as research subjects. This research was approved by REB at the University of Ottawa.

### Participants

Participants were invited to participate by different means of communication, including email-based invitation letters and paper-based posters. Any person who uses eCommerce and Movie RSs, and accepts to participate was included in the study. Our study respects participants’ privacy, such that none of their information (e.g., names and email addresses) is included or posted with the study. A total of 329 participants responded to the study. After filtering the responses (based on the measures described next), We retained a total of 279 responses. Table 3 summarizes the demographic information of the participants.

**TABLE 3:** Participants’ demographic information (*N* = 279).

Subject	Category	Count [percentage]	
Age	16–25	53	[19%]
	26–35	129	[46%]
	36–45	59	[21%]
	46+	38	[14%]
Gender	Male	177	[63%]
	Female	98	[35%]
	Undefined	4	[2%]
Continent	Asia	75	[27%]
	Europe	13	[5%]
	North America	186	[67%]
	South America	2	[1%]
	Australia (Oceania)	3	[1%]

## Data Analyses and Results

According to the Goal, Question, Metric (GQM) framework, which guided our evaluation, we conducted our analysis with several questions in mind to achieve the goals stated in *The Study*. These questions are:

• Q1: How do RS users’ characteristics (such as age, gender, culture, and personality traits) affect their responses to Cialdini’s persuasive principles?
• Q3: To what extent does a user’s responsivenessresponsiveness to different persuasive principles affected by the combination of the user’s characteristics and the recommender’s application domain?

The responses collected from this study (particularly the mean ratings) are used as metrics to answer the above questions. They are analyzed as follows: Demographic-related information is used to infer the impact of the user characteristics (namely age, gender, and culture) on the extent to which users got influenced by a particular persuasion. Answers to the Likert scale questions associated with the personality test are used to infer relationships between users’ personality types and persuasive principles. Finally, open-ended questions, where participants can write additional comments, are used to investigate unexplored insights on how users perceive different persuasive strategies.

To conduct statistical analysis of data, we used an open-source tool called JASP.^5^ JASP stands for **J**effrey’s **A**mazing **S**tatistics **P**rogram in recognition of the pioneer of Bayesian inference Sir Harold Jeffreys. It is a cross-platform and free of use statistics package. JASP helps in conducting statistical analyses through its easy-to-use GUI.

To determine whether the differences between groups are statistically significant, we performed the **An**alysis **o**f **Va**riance (ANOVA). ANOVA is used to analyze the differences between the means of multiple groups in a sample. In this study, groups are defined based on multiple factors, such as gender, age, and culture. For ANOVA analysis, the persuasive principles (i.e., users’ responsiveness to persuasive principles) were included as the dependent variable, while other factors (such as gender and age) were included as between-subject (or independent) variables. For data analysis the discuss one independent variable (such as the analysis in *User-Related, Context-Independent Analyses*), we used one-way ANOVA. While for other analysis that encompasses multiple independent variables, two-way ANOVA is used. The significance level (*α*) is set to be (0.05) for all ANOVA analyses, such that a *p-value* less than (0.05) is considered to be significant.

In addition to the ANOVA analysis, we also calculated the Effect Size (Lakens, 2013), which shows whether an intervention or experimental manipulation has an effect greater than zero or how big the effect is. Reporting the effect size is useful for various reasons (Lakens, 2013). First, it presents the magnitude of the reported effects in a standardized metric. Such standardized effect sizes help to communicate the practical significance of the results. Second, effect size compares standardized effect sizes across studies, which helps meta-analytic conclusions. Third, previously reported effect sizes (i.e., from previous studies) could feed the design of a new study. For instance, previous analysis can provide an indication of the average sample size needed for a new study. We deployed the Cohen’s *d*, one of the most widely used effect sizes (Cohen, 2013).

It is worth mentioning here that the focus of these analyses is to solely report on our findings regarding the four user-related factors, more than to provide explanations or justifications of each factor’s impact. Even social psychologists are trying to explain the relatively conflicted results originating from studies exploring the influence of persuasive principles. Based on our findings, we aim to develop general guidelines that could help tailor the development of RSs, with the user-related factors taken into considerations.

### Data Filtering

To ensure the reliability of the results, we filtered the responses based on different measures; First, we removed incomplete responses (where some questions are left unanswered). Twenty-three (23) responses were discarded after this step. Second, we considered the questionnaire’s completion time, which indicates whether participants have indeed read/answer the entire question or not. Based on a pilot study, we noticed that, on average, participants need about 20 min to complete the questionnaire fully. Hence, we discarded fifteen (15) responses that took less than 10 min. Third, some participants simply do not put in the effort required to respond accurately or thoughtfully to all questions asked. The data collected from such responses are known as careless or inattentive data (Curran, 2016). To detect and discard these responses, we used the attention questions technique. Attention questions (or items) are defined as “*Items placed in scale with explicit correct response*” (Curran, 2016). Particularly, we injected some irrelevant questions (i.e., questions that are not related to the context of the questionnaire) and clearly indicated what the participant should provide as an answer to this question. For instance, we associated the following text with one of the attention questions: “*You must give this question the rate 3*”). Responses from participants who got the attention questions incorrect were also discarded. Twelve records were dropped after this step. After these filtering steps, we retained 279 responses.

To ensure that the six cues representing Cialdini’s persuasive principles are reliably measured, we conducted McDonald’s omega (*ω*) reliability test using JASP tool. Mcdonald’s omega is suitable to measure internal consistency, and it has been proven as the best reliability statistics (Catalán, 2019). The value of (*ω*) ranges from 0 to 1, where a higher value represents better reliability. Supplementary Table S1 shows the (*ω*) results for the data. As the table indicates, the data about *Reciprocity* and *Scarcity* principles are highly reliable (with *ω* ≥ 0.7), and they are moderately reliable for the other principles.

### Descriptive Analysis

To evaluate RS users’ responsiveness to the six persuasive principles, we computed the overall mean rating (*μ*) for each principle. The overall mean rating is the average of all ratings received from the whole sample. The higher the mean rating, the more influence the principle. We found that, overall, participants perceived all principles as persuasive. As illustrated in Figure 2, all mean values are greater than the neutral value (i.e., Zero), which indicates that all strategies are influential. It can also be inferred from the figure that participants perceived *Social Proof* as the most influential strategy (*μ*= 3.03), followed by *Commitment* (*μ* = 3.0) and *Reciprocity* (*μ* = 2.96). On the other hand, *Scarcity* is perceived as the least influential strategy (*μ* = 2.12). These observations indicate that the majority of the participants believe that any of the six persuasive principles can influence their decisions to some extent. This is in line with the conclusion of Gkika and Lekakos (2014a), which indicated that accompanying persuasive explanations with a recommendation increases users’ acceptance of that recommendation.

**FIGURE 1:**
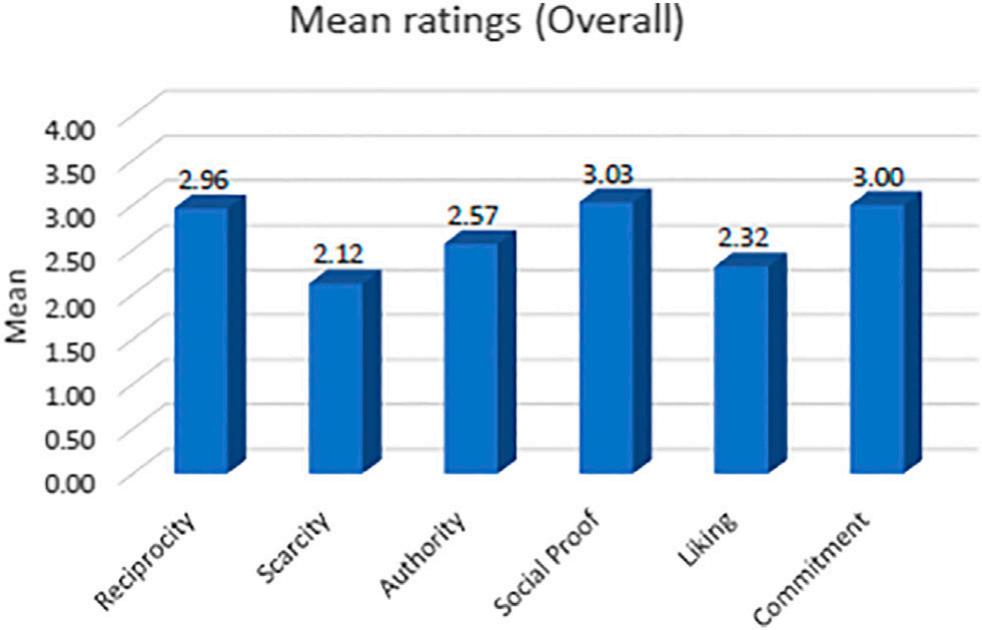
Mean rating values for the whole sample.

It is worthwhile to mention that these results, although they look low at first glance, they indicate an actual effect of the persuasive principles. That is because the study used a 7-points scale, with (−1) is the lowest value and zero is the neutral value (i.e., it indicates no effect). Since zero is the neutral value, any value more than zero indicates a persuasion effect. Besides, these statistics represent the whole sample. As described in the following sections, individuals vary in their responses to the persuasive principles. Therefore, each persuasive principle may persuade a group of users but not the others. These differences are the expected reasons behind these relatively average results. Hence, we can say that the mean values presented in Figure 1 reflect an important impact of the persuasive principles on the users. Despite the observations mentioned above, the particular question arises here: how the responses of RS users differ based on various factors, such as user-related factors as well as context-related factors? The two subsequent sections aim to answer this question.

### User-Related, Context-independent Analyses

This section focuses on the impact of user-related aspects/characteristics and how they affect users’ responsiveness to Cialdini’s persuasive principles. It discusses these factors in the separation of other aspects. Particularly, this section investigates how RS users differ in their reactions to the six persuasive principles based on four aspects: gender, age, culture, and personality traits, without considering the context at which users receive recommendations.

#### The Impact of Gender

**FIGURE 2:**
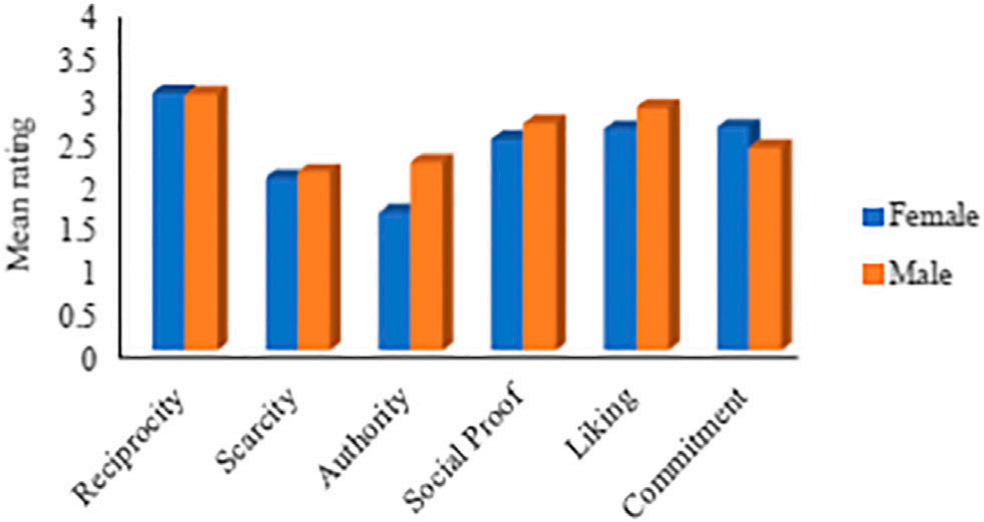
Mean rating based on gender (Female: 35%, Male: 65%).

The first aspect examined in this analysis is the users’ gender. Figure 2 compares the mean ratings of females compared to males. The figure shows that *Reciprocity* is the most effective principle for both genders. *Scarcity* and *Authority*, on the other hand, are the least effective for males and females, respectively. The figure also shows that males and females responses differently to all principles. However, the difference is marginal in regard to *Reciprocity* and *Scarcity*. Table 4 depicts the persuasion profiles (the order of the persuasive principles from the most persuasive to the least persuasive) for both gender groups.

**TABLE 4:** Persuasion profiles based on gender

	Most persuasive→				→Least persuasive	
Female	Reciprocity	Commitment	Liking	Social proof	Scarcity	Authority
Male	Reciprocity	Liking	Social proof	Commitment	Authority	Scarcity

The significance analyses (Supplementary Table S2) reveal that the difference is significant with respect to the *Authority* principle (F = 9.09, *p* = 0.003 ), with males being more persuadable than females. Cohen’s *d* shows that the effect size in regard to the *Authority* is close to moderate (Cohen’s *d* = 0.4). To a high extent, these results are in line with the findings of Oyibo et al. (2018a), who did not find gender differences with respect to *Reciprocity*, *Liking*, *Scarcity*, and *Social Proof*. Besides, the authors found a significant difference between males and females in terms of *Authority*, where males found to be more persuadable. Vargheese et al. (2020) also found that the effectiveness of the persuasive principles does not differ based on the gender of the participants.

#### The Impact of Age

**FIGURE 3:**
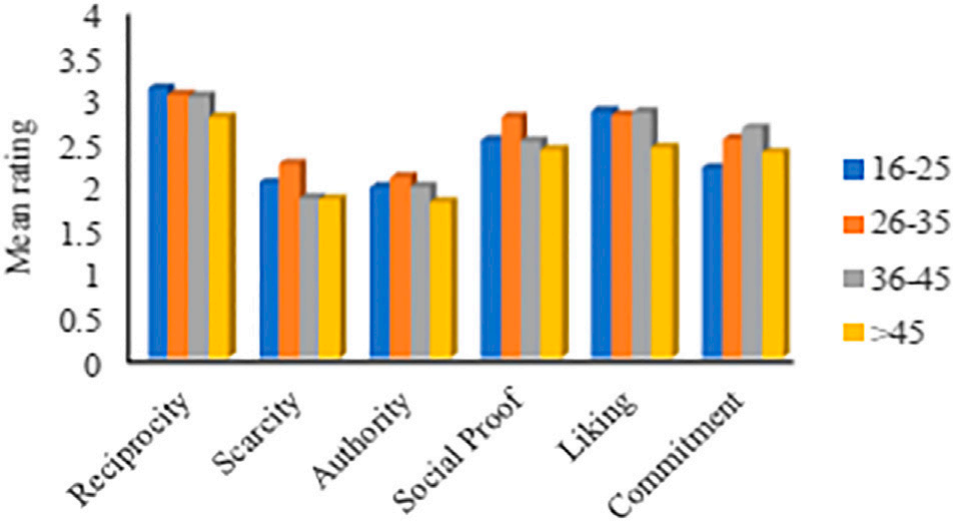
Mean rating based on age (16–25: 20%, 25–36: 40%, 36–45: 22%, >45: 18%).

This section investigates the effect of users’ age. Following the methodology of Aisha et al. (2018), we defined four age groups as follows: 16–25, 26–35, 36–45, and older than 45 years. Figure 3 shows the average ratings for the six persuasive principles for each age group. The figure shows some differences between the age groups, where participants of all ages agree that *Reciprocity* is the most influential principle. *Authority* is found to be the least influential for all groups, except the third group (ages 36–45), where *Scarcity* is found to be the least influential. Overall, younger users (ages 16–35) were found to be more susceptible to all principles except *Commitment*. This could be explained by the fact that younger people have less experience than older people. Therefore, they are generally influenced by multiple factors. On the other hand, older people have already gained enough experience and knowledge that help them be independent in their decisions. To emphasize the differences, Table 5 shows the differences in the persuasion profiles of the age groups.

**TABLE 5:** Persuasion profiles based on age.

	Most persuasive→				→Least persuasive	
16–25	Reciprocity	Liking	Social proof	Commitment	Scarcity	Authority
26–35	Reciprocity	Social proof	Liking	Commitment	Scarcity	Authority
36–45	Reciprocity	Liking	Commitment	Social proof	Authority	Scarcity
>45	Reciprocity	Liking	Social proof	Commitment	Scarcity	Authority

The significance test (Supplementary Table S3) shows no significant difference between users of different ages and how they got affected by all persuasive principles. This finding is in line with a recent study conducted by Oyibo et al. (2017a), who found that the differences between younger and older people are more significant in collectivist^6^ cultures than individualist^7^ cultures. Oyibo et al.’s findings support the results illustrated in Figure 3 since the majority of participants in this study are from individualist continents (as shown in Table 3). Also, a recent study by Vargheese et al. (2020) confirms these findings. Vargheese et al. stated that there is no actual relationship between the effectiveness of persuasive strategies and the ages of participants. Besides, studies in social sciences found that people’s personalities are relatively stable; they are stable throughout individuals’ lives, with some slight exceptions in the facets rather than the essential traits (Soto and John, 2012). Therefore, people’s decisions do not extensively change from one age to another.

#### The Impact of Culture (Continent)

Before analyzing the results based on the culture, it is worthwhile to mention that we categorized the responses based on the continents. That is, different cultures are distinguished according to the continent. As shown in Table 3, we received responses from five continents. However, since the number of participants from each of South America and Australia is very low (two and three participants, respectively), we omitted these responses from all culture-based analyses.

**FIGURE 4:**
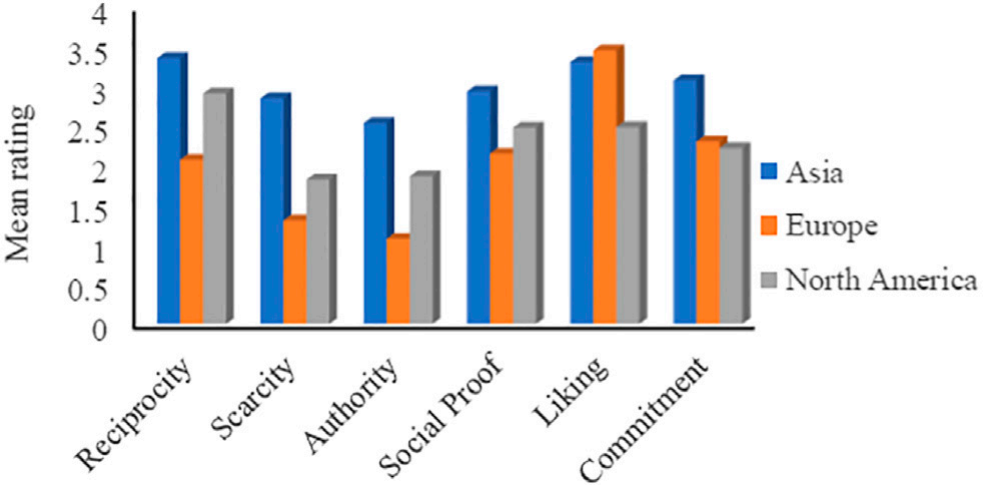
Mean rating based on the continent (Asia: 27%, Europe: 6%, N. America: 67%).

Figure 4 (respectively Table 6) depicts the mean ratings (respectively the persuasion profiles) of the three continents for the six principles. It shows that participants from all continents are differently susceptible to all principles, where the differences between the mean ratings are noticeable. According to the figure, Asians are the most susceptible to all principles except the *Liking* principle. On the other hand, Europeans are the least susceptible to *Reciprocity*, *Scarcity*, *Authority*, and *Social Proof*, while North Americans are the least susceptible to *Liking* and *Commitment*. Therefore, we can say that, overall, Asian participants were the most persuadable, followed by North Americans, and the Europeans were the least persuadable.

**TABLE 6:** Persuasion profiles based on culture groups.

	Most persuasive→				Least persuasive→	
Asia	Reciprocity	Liking	Commitment	Social proof	Scarcity	Authority
Europe	Liking	Commitment	Social proof	Reciprocity	Scarcity	Authority
N. America	Reciprocity	Scoial proof	Liking	Commitment	Authority	Scarcity

The significance test indicates a significant difference between the three groups in terms of all principles, as depicted in Supplementary Table S4. This is consistent with the results of Oyibo et al. (2017b), who indicated that culture has an impact on the difference between persons in terms of their responses to persuasive strategies. The effect size is found to be moderate in regard to the *Authority* principle and small in most other cases.

#### The Impact of Personality Traits

**FIGURE 5:**
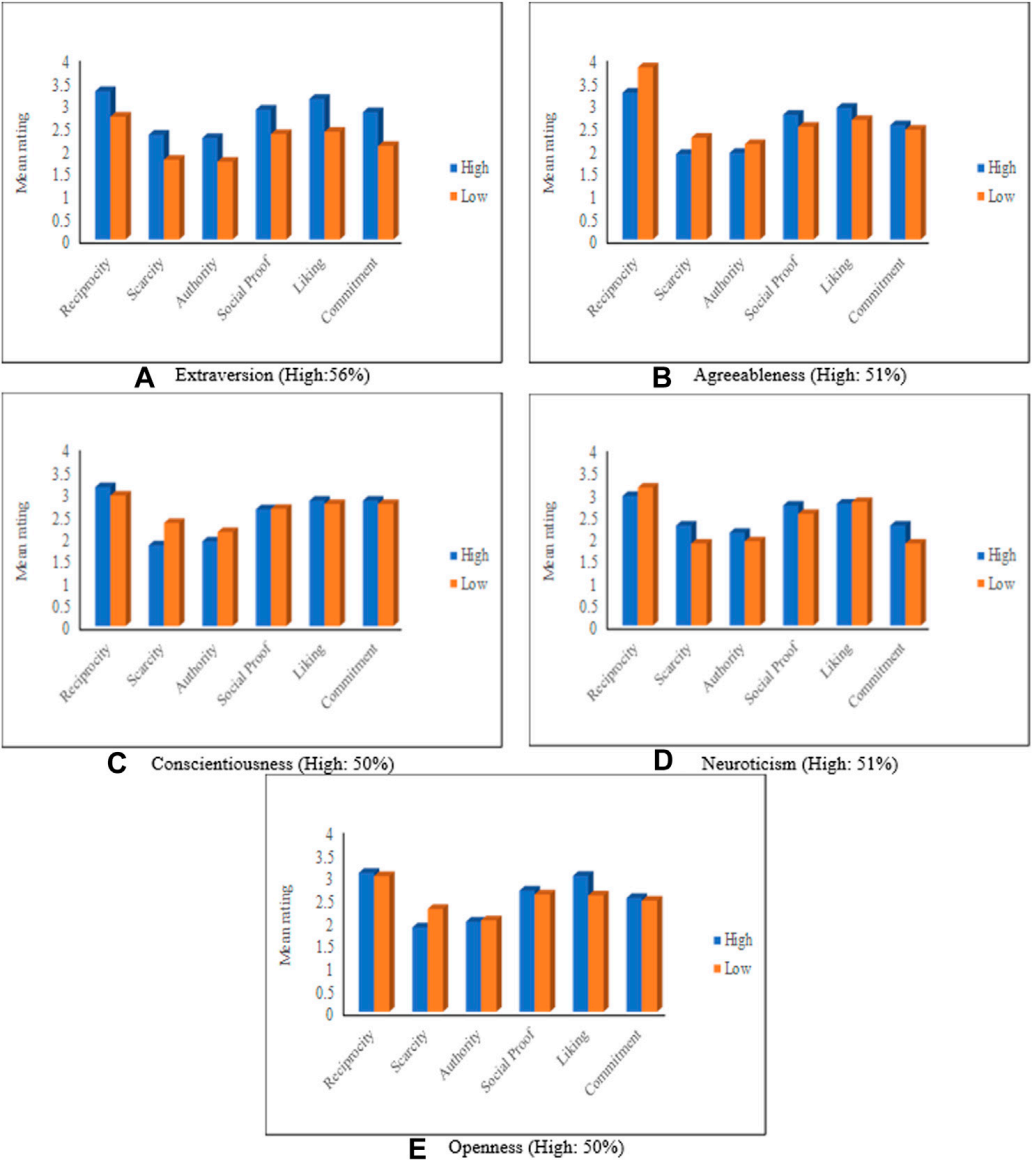
Mean rating based on the personality traits. **(A)** Extraversion, **(B)** Agreeableness, **(C)** Conscientiousness, **(D)** Neuroticism, **(E)** Openness.

This section discusses the relationship between the personality traits of RS users and Cialdini’s persuasive principles. Figure 5 depicts the average ratings for the persuasive principles grouped based on each of the five personality traits (discussed in *Personality Traits*), Table 7 shows the persuasion profiles, while Supplementary Table S5 depicts the significance analysis of these results. In this analysis, participants were categorized (based on the strength of their corresponding personality traits) into two groups, *High* and *Low*. High (respectively Low) means that the participant’s personality falls in the high (respectively low) end of the personality traits spectrum (where each personality trait can be considered as a spectrum graduating from a high degree to a low degree of that trait, as mentioned in *Personality Traits*). According to personality scientists, there are no cut-offs for personality traits. Typically, individuals’ traits are identified as relative to the whole sample. Thus, in this study, a person *Y* is considered high in a personality trait *PT* if Y’s score for trait *PT* is greater than the average score of all participants for that trait. Otherwise, *Y* is considered to be low in the *PT* trait.

**TABLE 7:** Persuasion profiles based on personality traits (H: high, L: Low).

	Most persuasive→					Least persuasive→	
Extraversion	H	Reciprocity	Liking	Social	Commit	Scarcity	Authority
	L	Reciprocity	Social	Liking	Commit	Scarcity	Authority
Agreeableness	H	Reciprocity	Liking	Social	Commit	Authority	Scarcity
	L	Reciprocity	Liking	Commit	Social	Scarcity	Authority
Conscientiousness	H	Reciprocity	Commit	Liking	Social	Authority	Scarcity
	L	Reciprocity	Liking	Commit	Social	Scarcity	Authority
Neuroticism	H	Reciprocity	Liking	Social	Commit	Scarcity	Authority
	L	Reciprocity	Liking	Social	Autority	Scarcity	Commit
Openness	H	Liking	Reciprocity	Social	Commit	Authority	Scarcity
	L	Reciprocity	Social	Liking	Commit	Scarcity	Authority

The statistical analyses performed on personality traits are done based on each personality. That is, for each personality trait, we analyzed the results based on the two groups (High, Low) of the traits. For the ANOVA analysis, the two groups of each personality were considered as the independent variables.

Figures 5A–E shows that mean ratings for each of the five personality traits are larger than t neutral value (Zero), which means that all personalities are susceptible to the six persuasive principles. The degree of responsiveness, however, varies from one personality trait to another and also varies within the same personality trait, depending on the level of that trait (i.e., high or low). The figure also shows that *Reciprocity* is the most influential principle for all personality types.

Figures 5A compares participants’ persuadability degrees based on their level of *Extraversion* trait. Participants who are high in extraversion are called *extroverts,* while low-level extroversion participants are called *introverts* (Rothmann and Coetzer, 2003). According to the figure, people who are high in extraversion are more susceptible to all persuasive principles than low extraversion people, with *Reciprocity* being the most influential principle and *Authority* being the least influential one. The responsiveness of high Extraversion people could be explained by their nature. That is, people who have high extraversion traits are more social and exposed to people. Therefore, they are open to hearing from others more than those who are low in extraversion traits. The significance test (Supplementary Table S5) indicates that both extraversion groups (i.e., High and Low) are significantly different (with *p* < 0.05) in their responses to all of the six persuasive principles. Also, the effect size is moderate for all principles.

Figure 5B reports on the results related to the *Agreeableness* trait. The figure shows that *Scarcity* is the least influential principle for *Agreeable* participants (i.e., who are high in *Agreeableness*), while *Authority* is the least influential principle for those who are low in *Agreeableness*. Moreover, *Reciprocity*, *Scarcity*, and *Authority* principles affect people who are low in aggreablenessmore than *Agreeable* people. The significance test shows that both Agreeableness groups are significantly different in their responses to *Reciprocity* (F = 7.23, *p* = 0.008) and *Scarcity* (F = 3.15, *p* = 0.07), where the effect size is found to be higher in regard to reciprocity (*d* = 0.33) comparing to Scarcity (*d* = 0.22).

Regarding the *Conscientiousness* trait, Figure 5C shows that *Reciprocity* is the most effective principle for both groups, while *Scarcity* is the least influential principle for people who are high in *Conscientiousness* and *Authority* is the least influential principle for people who are low in *Conscientiousness*. An expected reason behind these results is that a high level of conscientiousness indicates a preference for planned rather than spontaneous behavior. Thus, high conscientiousness people are not influenced by the scarcity principle because scarcity leads people to do things under pressure, which conflicts with the well-organized nature of conscientious people. It can also be noticed from the figure that the differences are marginal, especially in the term of *Social Proof*, *Liking*, and *Commitment* principles. However, the difference is significant in terms of *Scarcity* (F = 6.317, *p* = 0.013) with a low effect size (*d* = 0.3).

The *Neuroticism* personality trait is discussed in Figure 5D, which indicates that *Neurotic* persons (i.e., high in *Neuroticism*) are influenced the least by the *Authority* principle. On the other hand, those who are low in *Neuroticism* are influenced the least by *Scarcity* and *Commitment* principles. The figure also shows that *Neurotic* participants are more vulnerable to *Scarcity*, *Authority*, *Social Proof*, and *Commitment* than low-Neuroticism participants. According to the significance test, the responses of the two groups are significantly different in regard to *Scarcity* (F = 4.198, *p* = 0.04) and *Commitment* (F = 4.19, *p* = 0.04), with a small effect size for all principles

Finally, Figure 5E depicts the mean rating regarding the *Openness* trait. It can be noted from the figure that *Scarcity* and *Authority* are the least persuasive principles for *Open* persons (i.e., high in *Openness*) and *Closed* persons (i.e., low in *Openness*), respectively. Moreover, *Openned* people are more susceptible to *Reciprocity*, *Social Proof*, *Liking*, and *Commitment* principles compared to people who are low in Openness. The differences between both groups are minor in regard to *Reciprocity*, *Authority*, *Social Proof*, and *Commitment*, but they are significant in terms of *Scarcity* (F = 3.946, *p* = 0.48, *d* = 0.24) and *Liking* (F = 6.377, *p* = 0.012, *d* = 0.31).

To recap, the results provided in this section indicate that the extent to which Cialdini’s six persuasive principles influence RS users varies from one person to another based on their characteristics and traits and that each trait has a different impact on the degree of persuasion. The next section investigates another factor, namely the context (or the application domain). It discusses the impact of the context as a sole factor and the role it plays on the persuasion process when it is combined with the aforementioned user-related factors.

### Context-dependent Analysis

User-related aspects are not the only factors that affect the influence of a message (or recommendation); other factors such as the context are also important (Yoo et al., 2012; O’keefe, 2002). This section investigates the effect of the context on RS users’ acceptance of Cialdini’s six weapons of influence. The context is represented here by the *application domain*. Two recommendation domains were considered in this study, namely a movie RS and an eCommerce RS. The section begins by discussing the effect of the application domain in isolation of other factors. Then, it investigates the impact of users’ characteristics (discussed in the previous section) when the application domain is taken into account.

#### The Impact of Application Domain as a Sole Factor

**FIGURE 6:**
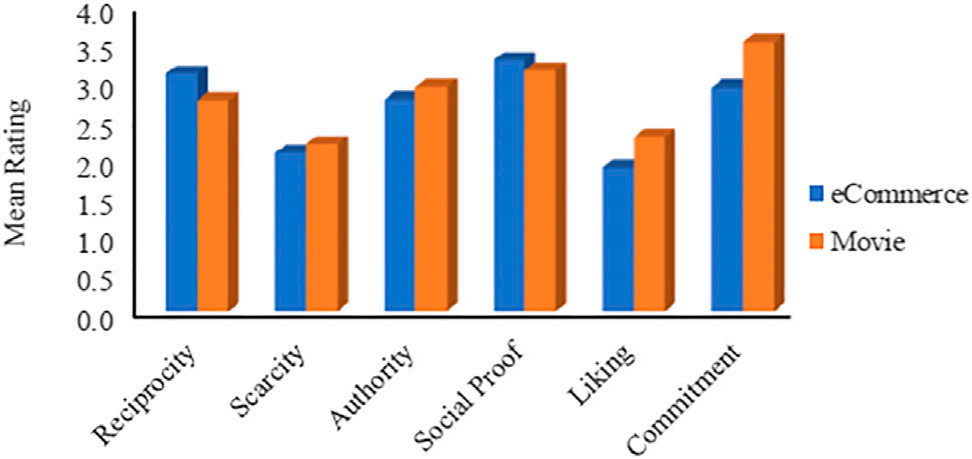
Mean rating based on the application domain.

This section reports how participants rated each persuasive principle in the eCommerce RS domain and the movie RS domain. Figure 6 depicts the mean ratings for each principle in both domains. It shows that all ratings are above the neutral value, which means that all principles influence users in both domains. The figure also shows that *Social Proof* and *Reciprocity* are the most and the second most influential principles in the eCommerce domain, while *Liking* is the least influential principle. On the other hand, the same users perceived *Commitment* and *Social Proof* as the most and the second most influential principles in the movie RS, and *Scarcity* as the least influential. From another perspective, the figure indicates that movie RS users are more vulnerable to persuasive principles than eCommerce RS users (with four out of six principles being more influential). One expected reason behind the higher persuadability in the movie domain is that watching a movie that might not be of users’ interest is less harmful than buying something that could not be of their interest. For instance, one of the participants stated, “…I will not pay money to buy anything unless I really need it … ”.

As mentioned previously, ANOVA analysis is used to examine the significance of differences in the results. The significance analysis results (Supplementary Table S6) indicate that the responses were significantly different in terms of three principles, namely *Reciprocity* (F = 16.78, *p* < 0.001), *Liking* (F = 17.56, *p* < 0.001), and *Commitment* (F = 40.68, *p* < 0.001), with effect size being moderate in terms of *Commitment* (*d* = 0.45) and small for the others Overall, the data shows that the application domain plays a role in affecting users’ sensitivity^8^ to the six principles.

#### The Impact of Gender in Combination With the Context

**FIGURE 7:**
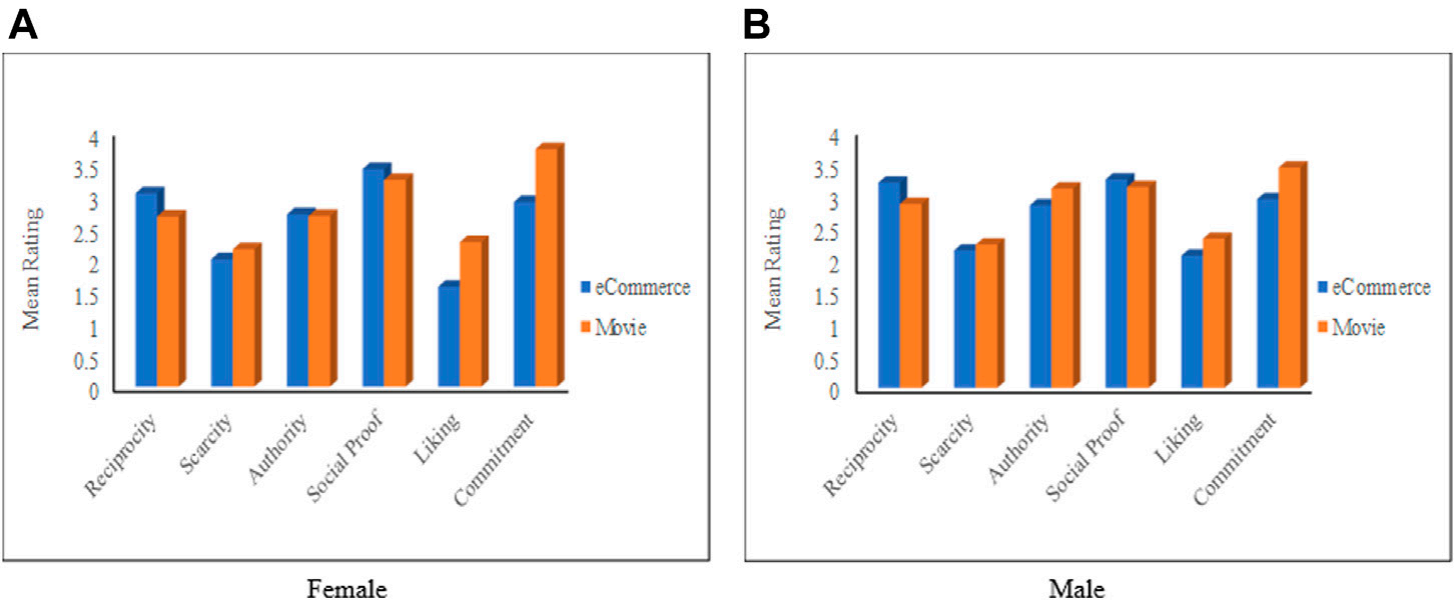
Mean rating based on gender for both application domains. **(A)** Female, **(B)** Male.

This section presents the results about how males’ and females’ responses vary from one domain to the other. Figure 7 depicts the average ratings for the six principles for both gender groups. Figure 7A indicates that females perceived *Reciprocity* and *Social Proof* as more persuasive in the eCommerce domain compared to the movie domain, while they perceived *Scarcity*, *Liking*, and *commitments* as more persuasive in the movie domain than in the eCommerce domain. The figure also shows that, in the eCommerce domain, *Social Proof* is the most influential principle for females, while *Liking* is the least influential principle. In the movie domain, however, the most influential principle is *Commitment*, whereas *Scarcity* is the least influential for females.

Regarding males participants, Figure 7B shows that *Reciprocity* and *Social Proof* are more influential in eCommerce than in the movie domain, while the other four principles are more influential in the movie domain. Besides, *Social Proof* and *Commitment* are perceived as the most influential principles in eCommerce and movie domains, respectively.

An important observation from Figure 7 is that the persuasion profiles^9^ of the same gender group are completely different from one domain to the other. Based on all the above results, we can say that the responses of females and males are affected by the application domain. The significance analysis (Supplementary Tables S7, S8) shows that the difference between users’ responses in different domains is statistically significant with respect to the *Reciprocity*, *Liking*, and *Commitment*, where *Reciprocity* is more influential in the eCommerce domain, while *Liking* and *Commitment* are more influential in the Movie domain. The Cohen’s *d* values show moderate effect sizes in terms of *Liking* (*d* = 0.43) and *Commitment* (*d* = 0.59).

#### The Impact of Age in Combination With the Context

**FIGURE 8:**
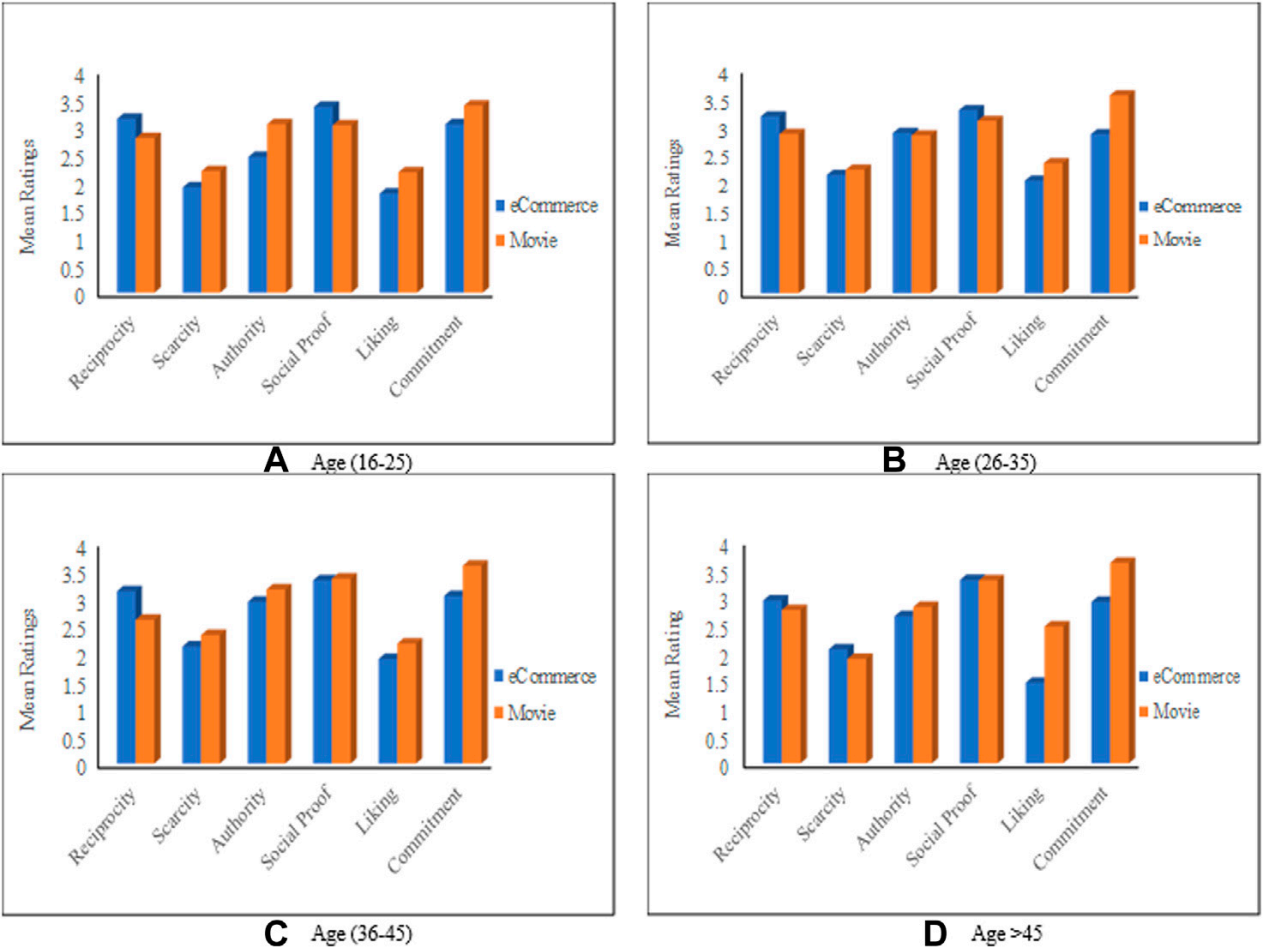
Mean rating based on the age and the application domain. **(A)** first age group (16-25 years old), **(B)** second age group (26-35 years old), **(C)** Third age group (36-45 years old), **(D)** Fourth age group (45 years and older).

This section discusses how RS domains affect users’ responses from different age groups to Cialdini’s persuasive principles. Figures 8A-D depicts each principle’s overall mean ratings for each age group based on the application domains. The figure contains four charts; each one summarizes the results regarding one age group. As the figure shows, the six persuasive principles are all influential in both domains (with mean values > zero) for all ages. Also, *Reciprocity* is perceived by all ages as more influential in the eCommerce domain. While *Liking* and *Commitment* are perceived as more influential in the movie domain for all age groups.

The figure also indicates that participants from all age groups perceived *Social Proof* as the most influential principle and *Liking* as the least influential in the eCommerce domain. On the other side, in the movie domain, *Commitment* is perceived as the most influential principle for all ages, while *Liking* is the least influential for groups (16–25, and 36–45), and *Scarcity* is the least influential for the others.

From Figure 8, we can also infer that the persuasion profiles for each age group are different from one domain to the other. For instance, Figure 8B shows that the persuasion profile of participants from the second group (i.e., age 26–35) in the eCommerce domain is (*Social Proof*, *Reciprocity*, *Authority*, *Commitment*, *Scarcity*, and *Liking*). However, the persuasion profile for the same group in the movie domain is (*Commitment*, *Social Proof*, *Reciprocity*, *Authority*, *Liking*, *Scarcity*), which completely different than the eCommerce persuasion profile. The above inference is true for all cases, where the persuasion profiles are different to a high extent from one domain to the other.

The ANOVA results (Supplementary Tables S9–S12) demonstrate that some of these differences are significant; First, the responses of users from the first age group (16–25) were significantly different in terms of *Reciprocity* (F = 4.78, *p* = 0.034, *d* = 0.27), *Authority* (F = 7.75, *p* = 0.008, *d* = 0.36), and *Commitment* (F = 3.864, *p* = 0.05, *d* = 0.23). *Reciprocity* is found more influential in the eCommerce domain, while *Authority* and *Commitment* are more influential in the Movie domain. Second, users of ages (26–35) responded significantly differently to *Reciprocity* (F = 5.65, *p* = 0.09, *d* = 0.24), *Liking* (F = 4.53, *p* = 0.035, *d* = 0.2), and *Commitment* (F = 19.79, *p* < 0.001, *d* = 0.5), with a small effect size in terms of *Reciprocity* and *Liking* and medium effect size in terms of *Commitment*. Third, the responses of the third age group (36–45) were significantly different with regards to *Reciprocity* (F = 11.17, *p* = 0.001, *d* = 0.45) and *Commitment* (F = 7.47, *p* = 0.008, *d* = 0.49). Finally, older users (45 years old and more) responded significantly differently to the *Liking* (F = 22.97, *p* < 0.001, *d* = 0.72) and *Commitment* (F = 11.19, *p* = 0.002, *d* = 0.64) principles.

Overall, Figure 8 shows that the responsiveness of users from the same age group to persuasive principles varies from one domain to the other. The most important observation in this context is that users of the same age respond differently to the persuasive principles if they interact with them in different contexts.

#### The Impact of Culture in Combination With the Context

**FIGURE 9:**
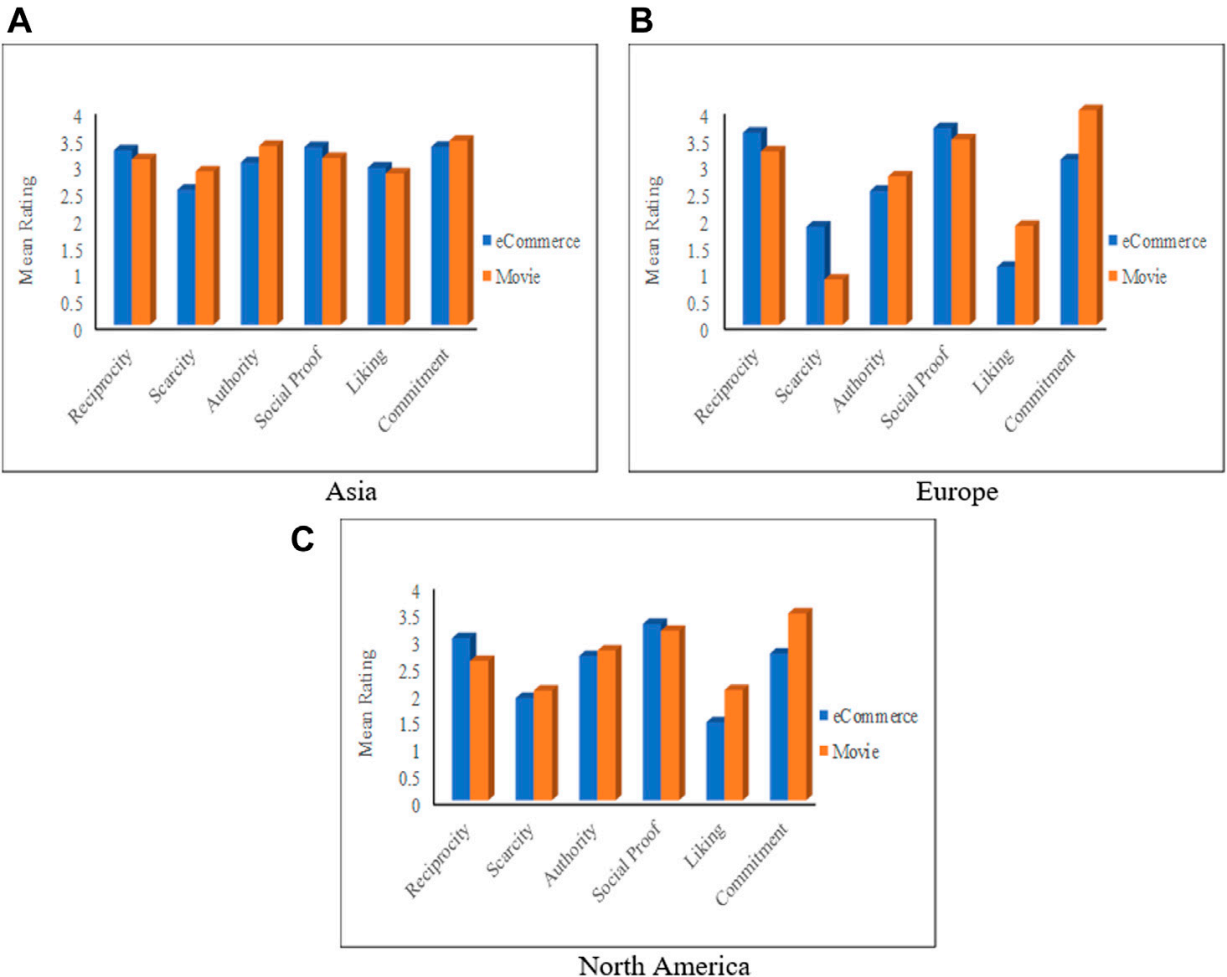
Mean rating based on the continent and the application domain. **(A)** Asia, **(B)** Europe, **(C)** North America.

The mean ratings of the six principles based on the culture and context are depicted in Figure 9. The first observation from this figure is that people from the three continents can be influenced by the six principles, but with some variations. *Commitment* is the most influential for Asians in both domains. This is because most Asian countries are collectivist; people in these countries focus on the group’s satisfaction. Therefore, they tend to be committed to their initial behaviors. On the other side, *Scarcity* and *Liking* are the least influential in the eCommerce and the Movie domains, respectively. These two principles are the least influential because, as mentioned above, Asian people are more focused on the group’s goals. Therefore, they are less affected by these two principles if they contradict their primary goal (i.e., the group).

For European and Northern Americans, *Social proof* and *Commitment* are the most influential in eCommerce and Movie domains, respectively, while *Liking* and *Scarcity* are the least influential in the eCommerce and the Movie domains, respectively. Figure 9 also indicates that the persuasion profiles of participants from one continent in the eCommerce domain are different to a very high extent from their persuasion profile in the movie domain. Particularly, persuasion profiles for North Americans are completely different (i.e., none of the principles occupies the same order in both profiles). The profiles of the Europeans are different except that *Authority* is the fourth most influential in both profiles. Finally, Asians’ profiles also vary from one domain to the other except for *Social Proof*, which is the third most influential in both domains.

The results of ANOVA analyses, depicted in Supplementary Tables S13–S15, show that Asians are the least affected by the context; the differences in their responses to the persuasive principles were significant for the *Authority* principle only (F = 4.239, *p* = 0.043), while the effect sizes were small (<0.22) for all principles. The Europeans responses were significantly different for both *Scarcity* (F = 6.76, *p* = 0.025, *d* = 0.66) and *Commitment* (F = 10.353, *p* = 0.008, *d* = 1.13) principles. Finally, the Northern Americans’ responses were significantly different in terms of three principles: *Reciprocity* (F = 17.494, *p* < 0.001, *d* = 0.31), *Liking* (F = 25.122, *p* < 0.001, *d* = 0.39), and *Commitment* (F = 45.007, *p* < 0.001, *d* = 0.57).

#### The Impact of Personality Traits in Combination With the Context

**FIGURE 10:**
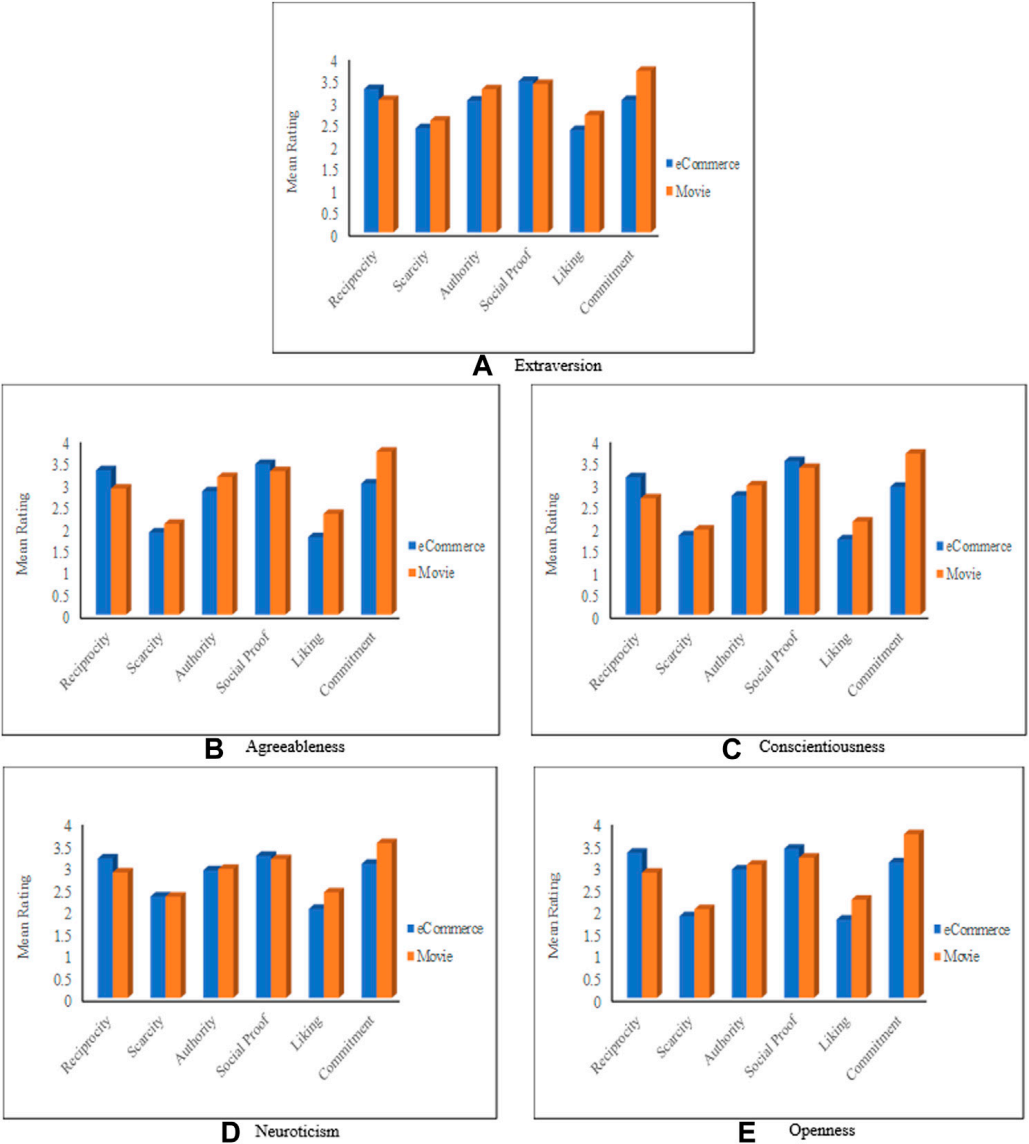
Mean rating based on the personality traits and the application domain. **(A)** Extraversion, **(B)** Agreeableness, **(C)** Conscientiousness, **(D)** Neuroticism, **(E)** Openness.

This section investigates how users with a particular personality trait perceived the persuasive principles in the eCommerce domain comparing to the Movie domain. Figures 10A-E depicts the average ratings for the persuasive principles based on the personality traits and the context. The figure contains five charts; each chart represents the results of one personality trait. For instance, Figure 10A shows the mean ratings given by the participants who are high in *Extraversion* traits. As a general observation, the figure shows that the means for all principles vary from one domain to another, which is true for the five personalities. We can also infer from the figure that in the eCommerce domain, *Social Proof* and *Liking* are the most and the least influential principles for all personalities, respectively. The Social Proof principle is very useful in eCommerce settings because, in such settings, the users deal with a wide range of alternatives, which they may not have enough information about. Thus, users are always seeking others’ opinions to decide. In the movie domain, however, *Commitment* and *Scarcity* are the most and the least influential principles, respectively. The dominance of *Commitment* in the movie domain is unsurprising because people usually show interest in a special type of movie. Therefore, telling the user that Movie-X belongs to the kind of movies she likes would have an impact on her decision.

The ANOVA analyses (Supplementary Tables S16–S20) demonstrated significant differences in the following cases: 1) Extroversion, Conscientiousness, and Neuroticism personalities perceived *Reciprocity*, *Liking*, and *Commitment* significantly different in the Movie domain compared to the eCommerce domain. These personality types perceived *Reciprocity* as more influential in the eCommerce domain, while *Liking* and *Commitment* are more influential in the Movie domain, 2) all principles, except *Social Proof*, were perceived significantly differently by *Agreeable* participants. *Reciprocity* is found more influential in the eCommerce domain, while the other four principles are found more influential in the Movie domain, 3) participants who have Openness personality perceived four principles significantly differently in the Movie domain compared to the eCommerce domain. These principles are *Reciprocity*, *Social Proof*, *Liking*, and *Commitment*. Medium effect sizes were found in terms of *Commitment* for all personalities, while a large effect size is found in terms of Liking for Agreeable people.

## Discussion and Design Guidelines

As mentioned earlier, the essential purpose of this study is to investigate the impact of Cialdini’s persuasive principles, when associated with recommender systems, on users of different personalities, ages, genders, cultures, and for different application domains. While conducting the study, our focus was, on one side, to report on the responses and ratings provided by participants regarding their experience with persuasive recommender systems (without providing justifications about the nature of the results). On the other side, based on the reported results, we aim to come up with empirical findings or insights, including several guidelines that could help in tailoring the design of persuasive RSs to users with different characteristics and also with taking different application domains into considerations. Despite the conservative nature of the obtained results, which might threaten their generalizability to actual persuasive recommender systems, we can still provide the following recommendations/guidelines for designers of such systems.

Considering Users’ Characteristics

• The persuasibility levels of users vary depending on their characteristics or personality features. Hence, we believe that tailoring the design of RS to individuals of different traits is a promising design direction that has the potential to improve recommender-user interaction.
• In this paper, the big five personality traits are taken as an example to distinguish the different personalities of users. Based on the results presented in *The Impact of Personality Traits*, the following insights are observed:
  o *Reciprocity* is the most influential principle for all personality types. So, it could be used as a unified strategy for all users in an application where users’ personalities cannot be obtained.
  o On the other side, *Authority* and *Scarcity* are the least or the second least influential principles for all personalities. Thus, they should be given the lowest priority in static (non-personalized) contexts.
  o Despite being applied in some applications, *Scarcity* is found to be the least influential strategy for *Agreeable*, *Conscientious*, and *Open* users. Having said that, designers are recommended to adopt a new style for attracting *Agreeable*, *Conscientious*, or *Open* users' attention, other than the threat of item’s scarcity.
  o All persuasive principles are more influential for low extraversion users compared with high Extraversion users.
• Gender, in general, is not a crucial factor to be considered alone when examining the impact of persuasive strategies on users (as discussed in *The Impact of Gender*). However, our study revealed that females and males respond significantly differently to the *Authority* principle, with males being more persuadable than females. Therefore, if the *Authority* principle will be used, we recommend using it with males more than females.
• According to the results presented in *The Impact of Age*, participants’ age does not significantly affect the persuadability process if considered in isolation of other factors.
• On the other hand, culture is a very important factor (as presented in *The Impact of Culture (Continent)*). RS users from different continents were significantly different in their responses to the persuasive principles, with Europeans being the most susceptible to the *Liking* principle and Asian are the most susceptible to all other principles

Considering the Context

• the results demonstrated that the application domain is an important factor to be considered. Our general recommendation is that if the RS seeks more personalized persuasive capabilities, the application domain should be considered. According to the results discussed in *Context-Dependent Analysis*, the following are some other designing tips related to the application domain and based on the domains under study:
  o The responses to the *Reciprocity*, *Commitment*, and *Liking* were significantly different. *Liking* and *commitment* are more effective in the Movie domain, while *Reciprocity* is more efficient in the eCommerce domain.
  o *Social Proof* can be used as a non-personalized strategy in the eCommerce domain, as it is found to be the most influential principle. On the other side, *Commitment* is the most influential principle in the Movie domain.

Overall:

• Associating persuasive strategies (Cialdini’s six persuasive principles, as an example in this work) with recommender systems shows, to a high extent, efficacy in impacting users’ decisions. This was clear by the mean rating for all principles in the two domains under study.
• Despite the aforementioned general tips, our study could not provide cutting-edge rules for deploying persuasive strategies in RSs. Also, as mentioned before, a one-size-fits-all design is not the most efficient approach for deploying persuasive systems. Therefore, our last insight is that an adaptive, personalized approach is required to provide the best persuasive experience for RSs.

### Threats to Validity

Before closing this discussion, it is important to highlight that the generalizability of our findings to a real RS may be threatened due to the limitation of our study; the main limitation stems from the fact that the study is based on a self-report questionnaire and not a real recommender system. A second limitation is that the diversity of the cultures is limited to three continents, with only 13 participants from Europe. This, in turn, may affect the generalizability of our findings to other populations. Accordingly, an important insight to be mentioned here is that the guidelines provided by our study and by the aforementioned related studies are general tips. These guidelines did not comprehend all possible design cases (i.e., the combinations of every factor with all other factors) and could not provide absolute or decisive rules for deploying persuasive strategies in RSs. Nonetheless, the study gives important observations and insights toward personalizing persuasive principles in the recommendation domain. Besides, and to mitigate these limitations, we plan to conduct a large-scale study that involves a bigger and more diverse sample of participants.

## Conclusion and Future Work

This paper elaborates on a user study conducted to examine the intersection between recommender systems, users, and persuasive principles. The paper reported on the results of how users of different characteristics (age, gender, culture, and personality traits) get influenced by (or interacted with) different persuasive principles that a recommender system deploys and how different contexts may affect users’ persuadability. The paper also suggested a set of design insights and guidelines as a takeaway for designers who wish to empower recommender systems with persuasive features to enhance their interaction with the users. The results demonstrated that 1) Cialdini’s principles could enhance the performance of RS if used properly. 2) Users’ personality traits are an important factor that could help in personalizing persuasive RSs. 3) Gender and Age are not as crucial as personality traits, especially if they are considered in isolation of other factors. 4) Culture is very important as it affects users’ decisions significantly. And 5) the application domain is an essential factor that should be taken into consideration.

Despite the work done in this study, the work is yet to continue in future. First, this study considered Cialdini's six principles of persuasion. Many other principles have also been proposed in the social science literature. So, we suggest studying the effect of other persuasive principles in the context of RS. Second, the results revealed that the application domain affects RS users’ vulnerability to different persuasive principles. Accordingly, a thorough study is required to explore the relationship (if any) between different persuasive principles and different domains of RSs. This study will provide the community with clear guidelines that help them deploy the right persuasive principles in the right recommendation domain. Finally, Yoo et al. (2012) suggest that the influence of a recommendation is affected by four main factors (the recommender, the recommendation, the user, and the context). Our study considered two aspects, namely user characteristics and the recommendation domain (the context). Further studies are still required to explore the effect of other factors, as well as the effect of the combination between these factors.

## Data Availability

The datasets presented in this article are not readily available because Based on the ethics approval granted to this research, the data should not be shared with anybody other than the authors. Requests to access the datasets should be directed to aalsl005@uottawa.ca.

## Footnotes

1 Five-Factor Model (FFM), and Big Five personalities (BF) will be used interchangeably through this document.

2 The term Persuadability indicates the influence level (or the persuasion ability) of the persuasive principle.

3 The term Persuadability indicates the influence level (or the persuasion ability) of the persuasive principle.

4
https://www.surveymonkey.com/

5 JASP team. JASP (version 0.10.2)[computer software]. See https://jasp-stats.org. Visited in June 2020.

6 A Collectivist culture (e.g., China, and Japan) is one that emphasize the needs of a group or a community over the individual.

7 An Individualist cultures (e.g., United States and Western Europe) emphasize personal achievement regardless of the expense of group goals.

8 The word “sensitivity” indicates how users’ are affected by the persuasive principles (i.e., the persuasiveness, or responsiveness to the persuasive principles).

9 Persuasion Profile represents the order of the persuasive principles from the most influential one to the least influential 0.
