# Supplementary material for: Users’ Responsiveness to Persuasive Techniques in Recommender Systems
Source: Front Artif Intell. 2021 Jul 8;4:679459. doi: 10.3389/frai.2021.679459 (PMC8297385; doi:10.3389/frai.2021.679459)
Supplement: Supplementary file 1 [file DataSheet1.docx]

**Supplementary Data Sheet**

**Supplementary Table S 1** Reliability Statistics (McDonald’s Omega)

|  | Reciprocity | Scarcity | Authority | Social proof | Liking | Commitment |
| --- | --- | --- | --- | --- | --- | --- |
| ω | 0.702 | 0.789 | 0.616 | 0.658 | 0.66 | 0.57 |

**Supplementary Table S 2** ANOVA significance test based on users’ gender.

|  | Reciprocity | Scarcity | Authority | Social proof | Liking | Commitment |
| --- | --- | --- | --- | --- | --- | --- |
| F | 0.019 | 0.108 | 9.095 | 1.086 | 1.911 | 1.397 |
| *p*-value | 0.892 | 0.742 | **0.003** | 0.298 | 0.168 | 0.238 |

**Supplementary Table S 3** ANOVA significance test based on users’ ages.

|  | Reciprocity | Scarcity | Authority | Social proof | Liking | Commitment |
| --- | --- | --- | --- | --- | --- | --- |
| F | 0.473 | 1.051 | 0.336 | 1.072 | 0.766 | 0.863 |
| *p*-value | 0.702 | 0.371 | 0.799 | 0.361 | 0.514 | 0.461 |

**Supplementary Table S 4** ANOVA significance test based on users’ culture.

|  | Reciprocity | Scarcity | Authority | Social proof | Liking | Commitment |
| --- | --- | --- | --- | --- | --- | --- |
| F | 6.114 | 12.194 | 7.375 | 3.565 | 10.842 | 7.986 |
| *p*-value | **0.003** | **<0.001** | **<0.001** | **0.03** | **<0.001** | **<0.001** |

**Supplementary Table S 5** ANOVA significance test based on users’ personality traits.

| Personality |  | Reciprocity | Scarcity | Authority | Social proof | Liking | Commitment |
| --- | --- | --- | --- | --- | --- | --- | --- |
| Extraversion | F | 11.836 | 7.698 | 7.591 | 10.446 | 18.418 | 15.812 |
|  | *p*-value | **<0.001** | **0.006** | **0.006** | **0.001** | **<0.001** | **<0.001** |
| Agreeableness | F | 7.235 | 3.154 | 1.012 | 2.476 | 2.443 | 0.27 |
|  | *p*-value | **0.008** | **0.077** | 0.315 | 0.117 | 0.119 | 0.604 |
| Conscientiousness | F | 1.333 | 6.317 | 1.174 | 0.003 | 0.192 | 0.192 |
|  | *p*-value | 0.249 | **0.013** | 0.28 | 0.96 | 0.662 | 0.662 |
| Neuroticism | F | 1.343 | 4.198 | 1.012 | 1.239 | 0.043 | 4.198 |
|  | *p*-value | 0.248 | **0.041** | 0.315 | 0.267 | 0.835 | **0.041** |
| Openness | F | 0.198 | 3.946 | 0.024 | 0.257 | 6.377 | 0.102 |
|  | *p*-value | 0.657 | **0.048** | 0.877 | 0.613 | **0.012** | 0.75 |

**Supplementary Table S 6** ANOVA significance test based on the application domain (the Whole sample)

|  | Reciprocity | Scarcity | Authority | Social proof | Liking | Commitment |
| --- | --- | --- | --- | --- | --- | --- |
| F | 16.781 | 1.237 | 2.009 | 3.254 | 17.568 | 40.688 |
| *p*-value | **<0.001** | 0.267 | 0.158 | 0.072 | **<0.001** | **<0.001** |

**Supplementary Table S 7** ANOVA significance test based on the domain & gender (Female)

|  | Reciprocity | Scarcity | Authority | Social proof | Liking | Commitment |
| --- | --- | --- | --- | --- | --- | --- |
| F | 5.746 | 0.566 | -20.5841 | 2.055 | 19.823 | 34.521 |
| *p*-value | **0.019** | 0.454 | 1.0 | 0.155 | **<0.001** | **<0.001** |

**Supplementary Table S 8** ANOVA significance test based on the domain & gender (Male)

|  | Reciprocity | Scarcity | Authority | Social proof | Liking | Commitment |
| --- | --- | --- | --- | --- | --- | --- |
| F | 10.52 | 0.541 | 2.701 | 1.171 | 4.649 | 15.225 |
| *p*-value | **0.001** | 0.463 | 0.102 | 0.281 | **0.032** | **<0.001** |

**Supplementary Table S 9** ANOVA significance test based on the domain & age (16-25 years old)

|  | Reciprocity | Scarcity | Authority | Social proof | Liking | Commitment |
| --- | --- | --- | --- | --- | --- | --- |
| F | 4.783 | 0.604 | 7.755 | 1.847 | 2.319 | 3.864 |
| *p*-value | **0.034** | 0.441 | **0.008** | 0.181 | 0.134 | **0.055** |

**Supplementary Table S 10** ANOVA significance test based on the domain & age (26-35 years old)

|  | Reciprocity | Scarcity | Authority | Social proof | Liking | Commitment |
| --- | --- | --- | --- | --- | --- | --- |
| F | 5.653 | 0.747 | 0.248 | 2.933 | 4.536 | 19.796 |
| *p*-value | **0.019** | 0.389 | 0.619 | 0.089 | **0.035** | **<0.001** |

**Supplementary Table S 11** ANOVA significance test based on the domain & age (36-45 years old)

|  | Reciprocity | Scarcity | Authority | Social proof | Liking | Commitment |
| --- | --- | --- | --- | --- | --- | --- |
| F | 11.174 | 1.277 | 0.748 | 0.044 | 1.475 | 7.476 |
| *p*-value | **0.001** | 0.263 | 0.391 | 0.835 | 0.23 | **0.008** |

**Supplementary Table S 12** ANOVA significance test based on the domain & age (45 years and older)

|  | Reciprocity | Scarcity | Authority | Social proof | Liking | Commitment |
| --- | --- | --- | --- | --- | --- | --- |
| F | 0.257 | 1.217 | 0.608 | 0.023 | 22.979 | 11.19 |
| *p*-value | 0.616 | 0.278 | 0.442 | 0.881 | **<0.001** | **0.002** |

**Supplementary Table S 13** ANOVA significance test based on the domain & culture (Asia)

|  | Reciprocity | Scarcity | Authority | Social proof | Liking | Commitment |
| --- | --- | --- | --- | --- | --- | --- |
| F | 0.973 | 2.638 | 4.239 | 1.801 | 0.392 | 0.298 |
| *p*-value | 0.327 | 0.109 | **0.043** | 0.184 | 0.533 | 0.587 |

**Supplementary Table S 14** ANOVA significance test based on the domain & culture (Europe)

|  | Reciprocity | Scarcity | Authority | Social proof | Liking | Commitment |
| --- | --- | --- | --- | --- | --- | --- |
| F | 1.158 | 6.76 | 0.022 | 0.288 | 1.669 | 10.353 |
| *p*-value | 0.305 | **0.025** | 0.884 | 0.604 | 0.223 | **0.008** |

**Supplementary Table S 15** ANOVA significance test based on the domain & culture (North America)

|  | Reciprocity | Scarcity | Authority | Social proof | Liking | Commitment |
| --- | --- | --- | --- | --- | --- | --- |
| F | 17.494 | 1.152 | 0.361 | 1.742 | 25.122 | 45.007 |
| *p*-value | **<0.001** | 0.285 | 0.549 | 0.189 | **<0.001** | **<0.001** |

**Supplementary Table S 16** ANOVA significance test based on the domain & Personality trait (Extraversion)

|  | Reciprocity | Scarcity | Authority | Social proof | Liking | Commitment |
| --- | --- | --- | --- | --- | --- | --- |
| F | 5.209 | 1.677 | 3.132 | 0.445 | 6.404 | 30.704 |
| *p*-value | **0.024** | 0.197 | 0.079 | 0.506 | **0.012** | **<0.001** |

**Supplementary Table S 17** ANOVA significance test based on the domain & Personality trait (Agreeableness)

|  | Reciprocity | Scarcity | Authority | Social proof | Liking | Commitment |
| --- | --- | --- | --- | --- | --- | --- |
| F | 10.041 | 1.509 | 4.217 | 1.595 | 18.547 | 33.654 |
| *p*-value | **0.002** | **0.001** | **0.042** | 0.209 | **<0.001** | **<0.001** |

**Supplementary Table S 18** ANOVA significance test based on the domain & Personality trait (Conscientiousness)

|  | Reciprocity | Scarcity | Authority | Social proof | Liking | Commitment |
| --- | --- | --- | --- | --- | --- | --- |
| F | 11.453 | 1.044 | 2.343 | 1.535 | 11.192 | 35.362 |
| *p*-value | **<0.001** | 0.309 | 0.128 | 0.218 | **0.001** | **<0.001** |

**Supplementary Table S 19** ANOVA significance test based on the domain & Personality trait (Neuroticism)

|  | Reciprocity | Scarcity | Authority | Social proof | Liking | Commitment |
| --- | --- | --- | --- | --- | --- | --- |
| F | 8.714 | 0.277 | 0.023 | 0.666 | 5.821 | 10.961 |
| *p*-value | **0.004** | 0.6 | 0.88 | 0.416 | **0.017** | **0.001** |

**Supplementary Table S 20** ANOVA significance test based on the domain & Personality trait (Openness)

|  | Reciprocity | Scarcity | Authority | Social proof | Liking | Commitment |
| --- | --- | --- | --- | --- | --- | --- |
| F | 16.605 | 0.665 | 0.477 | 3.183 | 17.391 | 24.659 |
| *p*-value | **<0.001** | 0.416 | 0.491 | 0.077 | **<0.001** | **<0.001** |
